# The Translation of Intergenerational Care Potential Into Care Receipt of Older Parents: A Prospective Study

**DOI:** 10.1177/01640275251326507

**Published:** 2025-03-14

**Authors:** Ying Shen, Theo G. van Tilburg, Mariska van der Horst

**Affiliations:** 1 Department of Sociology, 1190 Vrije Universiteit Amsterdam, Amsterdam, The Netherlands

**Keywords:** caregiving, family structure, intergenerational relations, longitudinal methods, care potential

## Abstract

This study prospectively examined the extent to which intergenerational care potential translated into parent’s care receipt. Data were from 510 parents (aged 70–97 years at baseline) who reported on their 1496 adult children in the Longitudinal Aging Study Amsterdam, with seven observations over ten years. Joint care potential considered the number of children and their care potential types. Children with high care potential lived nearby, had frequent contact, and had significant emotional and instrumental support exchanges with their parent. For unpartnered parents, each additional child increased the likelihood of receiving intergenerational care. Having children with high care potential further increased this likelihood. For partnered parents, receiving care was more likely if all children had medium or high care potential; an additional child only contributed under this condition. Policies and practice should not assume that older parents will receive care solely based on having multiple children or a child living nearby.

## Introduction

Recent reforms in long-term care policy have put families in the spotlight of care provision, for example in the Netherlands (Maarse & Jeurissen, 2016). The Latent Kin Matrix suggests that family members have great potential to provide care and support, even if they are not currently involved in caregiving or other direct assistance (Riley & Riley, 1993). Family relationships are often not static but have the capacity to change depending on needs, such as the onset of illness. For example, 46% of older adults experienced a change in their child caregivers over the course of two years (Szinovacz & Davey, 2013). Therefore, evaluating the support resources for older adults should go beyond identifying current caregivers to include those with caregiving potential that could be activated. Usually, adult children’s availability is dormant and there is a potential to respond to parental needs (Silverstein et al., 1997). The study aims to examine this latent reserve as intergenerational care potential: we explore its characteristics by showing the extent to which it evolves into future care for older parents.

The Latent Kin Matrix indicates that the support that family members offer each other depends on “the predispositions of each individual and the continuing motivation to negotiate and renegotiate their joint lives” (Riley, 1983, p. 446). Many studies have investigated which child is more likely to provide care, focusing on the characteristics of the child and the parent using a dyadic approach. For example, Stuifbergen et al. (2008) reported that motivation of care and socio-demographic characteristics of both the child and parent correlate to caregiving. Bui et al. (2022) found path dependence: children who were previously supportive were most likely to provide care. In addition to such individual indicators, studies considered within-family dynamics by incorporating multiple intergenerational relationships and parental expectations into the analysis. Vergauwen and Mortelmans (2021) found that a child was more likely to provide care if they were closer to their parents than their siblings in terms of distance and contact and if they were daughter with brothers rather than sisters. Leopold et al. (2014) found that an individual children’s transition in caregiving was influenced by their sibling’s relative costs (e.g., geographic distance) and relative commitments (e.g., birth order and parental expectation).

Another body of research investigated whether an older parent received care not from an individual child, but from the group of children. According to the configurational approach (Widmer, 2016), family dynamics operate at both the level of individuals, shaped by their own opportunities (e.g. contact frequency) and constraints (e.g. geographic distance), and the structural level with mutual dependencies among family members (e.g., care division). This approach considers the family network as a whole, where individuals are nested and interdependent. It has been applied in research on intergenerational care for older adults. Parents were more likely to receive intergenerational care if they had more children and at least one daughter (Grundy, 2005; Grundy & Read, 2012). A higher proportion of children who were daughters, biological children, and had no minor children predicted a higher proportion of children providing care (Lin & Wolf, 2020). Having more children and more daughters than sons predicted receiving care from children rather than from a partner or no care at all, and parents with only sons tend to receive formal care (Batur et al., 2024).

In the current study, we adopted the configurational approach to examine whether older parents received intergenerational care. We went beyond counting the number or proportion of children with a specific characteristic by describing – building on previous work – the care potential of the group of children. First, we developed a typology of *relational care potential* within the parent-child dyad. Based on the Intergenerational Solidarity Model (Bengtson & Roberts, 1991) and the discussion on the latent form of intergenerational cohesion by Silverstein et al. (1997), we use the opportunity, association, and function dimensions to assess the strength and potential of individual parent-child ties. Then, we aggregated the types of individual parent-child ties (dyad level) to describe the *joint care potential* of the group of children (family level). The joint care potential includes the number of children as a structural characteristic, extended by the composition of the children’s *relational care potential* types. Third, we used a prospective longitudinal design to determine how joint care potential affects future parental care receipt.

We defined care as any personal care or help with transportation as well as household, nursing, and administrative tasks. Although care potential may work out differently for various types of care, we combined a wide range of care types to provide a comprehensive overview of intergenerational care receipt. We distinguished between unpartnered and partnered parents when examining intergenerational care because partners – when available – are typically the primary caregivers (Szinovacz & Davey, 2013).

### Relational Care Potential of Individual Children

Intergenerational Solidarity Model (Bengtson & Roberts, 1991) captures different dimensions of the strength of parent-child ties. Based on this model, developing a typology of *relational care potential* helps to reflect the potential of specific children in providing care for parents. Silverstein et al. (1997) distinguished five types of intergenerational relationships such as a “tight-knit” latent class (children high on closeness, contact frequency, and support exchange, and with small geographic proximity) and an “intimate but distant” class (children high on closeness but at great geographical distance). The number of indicators used varies across studies, which may be related to the number of types found; geographic proximity, frequency of contact, emotional closeness, affection or quality, and exchange of support are often included as indicators (Hwang et al., 2022; Kim et al., 2020; van Houdt, 2023; Wang et al., 2023; Zeng & Li, 2024). Typically, the latent class analysis was used to construct the typology.

In this study, we used four indicators that cover three dimensions of the Intergenerational Solidarity Model to assess a child’s care potential. Our dataset does not allow us to explore all the indicators of care potential proposed in the literature. *Geographic proximity* represents the opportunity structure, promoting children’s care provision by reducing the cost of time and energy for traveling (Dykstra & Fokkema, 2011) and facilitating frequent visits. It enables children to react responsively to care needs that parents are unable or unwilling to articulate remotely, or that can only be determined visually or in person. The shorter the distance of travel, the more likely children are to provide care (Pillemer & Suitor, 2014) and to be the primary caregiver (Szinovacz & Davey, 2013). *Frequent contact* represents the association solidarity**,** facilitating caring (Grundy & Read, 2012). For example, children are more likely to be aware of their parents’ needs if they call or visit their parents often (Dykstra & Fokkema, 2011), which is related to the opportunity structure. The exchanged *emotional* and *instrumental support* reflects functional solidarity and indicates children’s propensity for providing care due to intergenerational reciprocity (Grundy, 2005; Leopold et al., 2014). Furthermore, support of children is likely to continue when their parents have increased care needs due to the parent’s path dependency and children’s developing commitments, that is, the child who assumed the role of caregiver would feel increasingly obligated to provide care (Leopold et al., 2014).

The gender of children also appears to be important. Daughters generally provide more frequent, intensive, and diverse intergenerational care than sons (Schmid et al., 2012). This gender difference fits with traditional gender ideology, where the availability and aptitude of daughters is seen as greater than that of sons (Grunow et al., 2018). Inequality between men and women in the division of childcare and domestic work as well as the gender pay gap persists over time (Yerkes et al., 2020).

### Joint Care Potential of the Group of Children

The joint care potential of a group of children consists of both the number of children – not taking into account their care potential – and the composition of children’s relational care potential. The number of children is the first indicator of intergenerational care potential (Bengtson & Roberts, 1991). The more siblings a child has, the less likely they are to provide care (Vergauwen & Mortelmans, 2021). If there are other children, the need to provide care for an individual child is lower and arguments may arise between siblings over the distribution of care, which reduces the motivation to provide care. But at the family level, parents with more children are more likely to receive care, because there are simply more children that can provide care. For example, an older single-child parent may not receive intergenerational care when the child has competing tasks or is in poor health. Previous studies found a positive association between the number of children and the likelihood of receiving intergenerational care. Yet, findings varied in whether each additional child or a minimum number of children is needed to increase this likelihood (Grundy & Read, 2012; Sun, 2002). Our first research question is: (1) Does parents’ intergenerational care receipt depend on the total number of children, and if so, what is the optimal number?

Having children who have many favorable characteristics, including living nearby, visiting their parents often, and having a history of exchanged support, may ensure that care is provided to a parent who needs it. However, it is unknown whether having a ‘tight-knit’ relationship with one child (i.e., one child having all favorable characteristics) is sufficient, whether more ‘tight-knit’ children are necessary, or whether their favorable attributes can be distributed across different parent-child relationships. An example of the latter scenario is a parent with one child who is high in opportunities but low in exchanging support, and another child, despite living at a distance, provides emotional and instrumental support by making calls and organizing tasks online. Szinovacz and Davey (2013) and Vergauwen (2024) examined family dynamics and found attrition and replacement among adult children who provided care over time. This may be related to a child’s responsibilities (e.g. caring for one’s own children), incompatibility with paid work, or an increasing care burden. This suggests that having multiple children with medium or high care potential increases the likelihood of older parents’ receipt of care. Our second research question is: (2) How does the quality (care potential) of children, combined with their quantity (number), contribute to a parent’s likelihood of receiving intergenerational care? In answering this question, we examined a threshold model and an accumulative model (Suitor et al., 2018). Specifically, we examined whether having a child with high care potential is essential, or whether medium care potential is sufficient. Additionally, we considered whether having more children with a particular (high or medium) care potential increases the likelihood of receiving care compared to having just one child with that care potential.

### Contextual Factors Associated with Intergenerational Care Receipt

Except the joint care potential of children, contextual factors such as norms towards informal and formal care, parent’s care need, use of alternative care options potentially influence the likelihood of parent’s care receipt from children. Considering them takes into account alternative explanations and reduces omitted bias.

#### Norms towards Informal and Formal Care

Children’s moral obligation to provide care is known as filial responsibility expectations (van der Pas et al., 2005). Parent’s expectations and norms could be perceived by children through informal conversations in their daily lives, motivating children to participate in care provision when it is needed (Hamon & Blieszner, 1990). Parent’s norms towards informal and formal care are regarded as a type of normative belief about the care responsibility of family and government (Jacobs et al., 2018).

#### Care Need

The incidence of care need has been regarded as the starting point of caregiving behaviors (Broese van Groenou & de Boer, 2016). Care need impacting care receipt has been specified in different ways, such as a decline in self-reported health (Chen et al., 2021), the incidence or increase of functional and cognitive impairments (Pezzin et al., 2015), increased frailty (Lin & Wolf, 2020), losing one’s partner (Silverstein et al., 2006), and a higher chronological age (Grundy, 2005).

#### Use of Alternative Care Options

If parents receive care from alternative resources, the translation from intergenerational care potential to parental care receipt may not happen. Previous studies examined the impact of use of informal care from social networks (Tolkacheva et al., 2011), formal care (Motel-Klingebiel et al., 2005), and privately paid care (Jacobs et al., 2018) on the receipt of intergenerational care.

## Method

### Sample

Data were obtained from the Longitudinal Aging Study Amsterdam (LASA) (Hoogendijk et al., 2020). Samples of men and women were taken from the population registers of three Dutch cities and six surrounding small municipalities. We used the 2011–2012 follow-up observation as baseline (T_0_), because comprehensive information on care receipt was collected from this observation. The criteria for selecting respondents and observations are illustrated in Figure S1 in the Supplement. Our final sample included 510 individuals from different households: 202 males and 308 females, of whom 486 were born in the Netherlands. At baseline, ages ranged from 70 and 97 years (M = 78; SD = 5). 196 individuals had no co-resident partner, while 314 lived with their partner at baseline. They had a total of 1496 adult children (M = 2.9; range 1–10), and reported gender and relational characteristics of each of their children. We included six follow-up observations (T_1_-T_6_), with mean intervals of 3.9 (*N* = 510), 4.7 (*N* = 449), 5.4 (*N* = 413), 6.1 (*N* = 387), 6.9 (*N* = 353) and 10.0 (*N* = 237) years since baseline. This resulted in 2859 observations among 510 respondents included in our final analyses.

### Measures

#### Intergenerational Care Receipt

Respondents were asked if they received any personal care, transportation assistance, or help with household, nursing, and administrative tasks. For example, a question was: “Are you currently receiving help with household tasks?” Subsequent questions asked about the type of caregivers. Intergenerational care was identified when parents indicated that they had received care from children.

#### Children

Information about respondents’ children was collected at various observations (van der Pas & van Tilburg, 2010): about travel time between parents and children (in minutes; we recoded values into eight classes), how often they had contact (because of the small numbers, we merged four categories into “once a month or less”), and how often instrumental and emotional support was exchanged (1 = never; 2 = sometimes; 3 = seldom, 4 = often; we averaged given and received). Values with a frequency of less than five per cent were top- or bottom-coded. We took the last reported gender of child in our analyses.

#### Norms towards Informal and Formal Care

Two questions measured norms towards informal care (*r* = 0.41; e.g. ‘If you need temporary help, you should be able to ask your children, family or neighbors’); two questions were about formal care (*r* = 0.46; e.g. ‘It’s annoying to be dependent on professional agencies for help’), and their scores were reversed. For both sets, we calculated the average scores, with higher scores indicating more positive norms. Filial responsibility expectations were measured with 16 items (van der Pas et al., 2005), for example, ‘Children should be willing to give up free time for their parents.’ Cronbach’s alpha = 0.86. Response options ranged from 1 = completely disagree to 5 = completely agree.

#### Care Need

Respondents reported if they had any of seven chronic diseases. We counted the number. Self-perceived health was a rating from 1 = very good to 5 = poor. Physical impairments were self-reported for six activities of daily living, for example, walking up and down fifteen steps (1 = no difficulties to 5 = cannot do it at all). Cronbach’s alpha = 0.83. Cognitive impairments were measured by a shortened version of the Mini-Mental State Examination (0 = excellent to 16 = poor) (Folstein et al., 1975). Values with a frequency of less than five per cent were top-coded. Age was measured as chronological age. Correlations among these five indicators ranged from 0.06 to 0.46. Additionally, respondents who lost their partner since baseline were categorized as 1; those who had not were categorized as 0.

#### Use of Alternative Care Options

We categorized care providers into the following groups: partner (if applicable), informal caregivers (including other household members, children-in-law, other family members, neighbors, friends, and volunteers), publicly paid care (such as district nurse and home care professionals), and privately paid care.

#### Other Control Variables

We included parents’ gender, net monthly household income (categorized into twelve classes; we took the mid-point values), educational level in years (ranging from 5 = incomplete elementary schooling to 18 = completed higher education) at baseline, and whether they were stepparents at baseline.

### Procedure

We used Latent Class Analysis (LCA) to identify the typology of relational care potential at baseline, based on four indicators: geographic proximity, contact frequency, emotional exchange, and instrumental exchange. To select the optimal model, we used the Bayesian Information Criterion (BIC) and the likelihood ratio chi-square goodness of fit test (L^2^) (Nylund et al., 2007). Additionally, we looked at entropy to check the classification accuracy of the model and class sizes. Latent Gold (Vermunt & Magidson, 2000) was used.

We aggregated relational care potential classes into joint care potential by assessing the number of children in each class identified through LCA (as illustrated in Figure S2). Then, joint care potential was used to predict parent’s care receipt through multilevel logistic regression, with observations nested within respondents. We took a stepwise approach. Model 1 included time since T_0_ to account for the longitudinal structure. Model 2 added the total number of children. Next, we added the number of children in each relational care potential class. To address the dependence on the total number of children, we added whether having a child (if possible, also having a second child) in specific classes one by one in the model**.** We tested nine Models 3, that is, different combinations of care potential classes, and retained classes that were statistically significant. In Model 4, we controlled for children’s gender (i.e., having at least one daughter vs. only sons) and its interaction with parental gender (Grigoryeva, 2017). Model 5 added parent’s characteristics, their norms, care need, and received care from others. In a robustness check, we examined whether the predictive effect of joint care potential differed when cross-sectional data were excluded. We stratified all regression analyses by respondent’s partner status at baseline because a partner might be the primary caregiver and might decrease the need for intergenerational care. Information on missing values is outlined in Table S1.

## Results

### Description of Care Potential

We selected the LCA solution with five classes (model fit is presented in Table S4). We labeled classes based on discussions in the literature or their attributes on four characteristics (Table S5). Children in Class A, labeled as the *tight-knit* (Silverstein et al., 1997), lived near their parents, had frequent contact with them, and exchanged emotional and instrumental support frequently. Children in B, labeled as the *sociable* (Silverstein et al., 1997), typically lived relatively close to their parent (i.e., within 20 minutes) but had only occasional emotional exchange. Children in C, labeled as the *remote and emotionally connected*, typically lived farther away but had frequent emotional exchanges. Children in class D were labeled as the *remote and staying in touch* (Wang et al., 2023)*,* and those in Class E were labeled as the *detached* (Silverstein et al., 1997). Children in A have high care potential due to their predominant advantages across all four characteristics. Those in classes D and E have low care potential because of their predominant disadvantages in three out of four characteristics. Classes B and C exhibit a more balanced mix of advantages and disadvantages, positioning them as having medium care potential. The share of daughters was high in classes A and C (61% and 58%, respectively) compared to the other classes (ranging from 38% and 42%).

Moving to the parent level, 35% of them had a child in class A, 56% had no children in class A but at least one child in classes B or C, and the remaining 9% had all children in class D or E. Because few respondents had many children, we did not distinguish in the number of children between five and more, in classes A and E between one or more, and in classes B, C, and D between two or more children. The five variables of the classes are generally weakly and negatively correlated (−0.26 ≤ *r* ≤ 0.04). They correlate between 0.21 (class A) and 0.47 (class B) with the number of children.

### Description of Intergenerational Care Received and Control Variables

When parents received intergenerational care, it often involved assistance with arranging help or aids, with home modifications, and with financial matters (68%), as well as help with trips, visits, and access to healthcare (60%). Additionally, 31% of the parents received help with household tasks, such as preparing meals, shopping, and cleaning. However, they rarely received help with personal care (2%) or nursing (1%).

**Figure 1.**
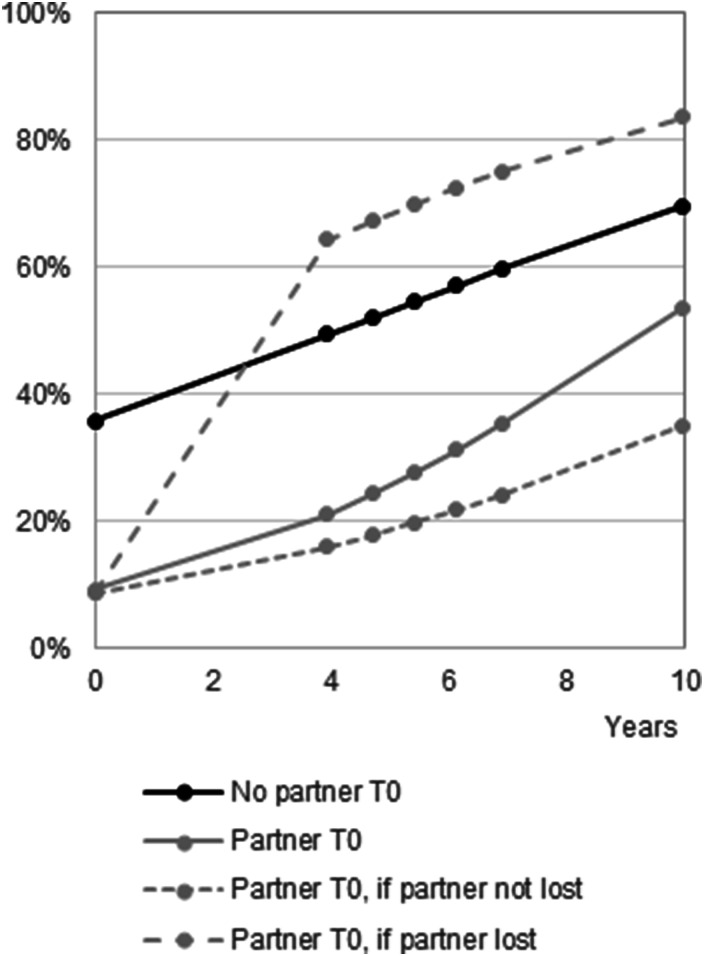
Likelihood of care received from children over ten years. *Notes:* 237 ≤ *N* ≤510. T_0_-T_6_ are marked by dots. Predicted probabilities of parents having a partner at T_0_ who lost their partner are given for those who lost their partner between T_0_ and T_1_.

More unpartnered parents received intergenerational care than partnered parents. The percentage of unpartnered parents who received intergenerational care increased from 36% at T_0_ to 70% at T_6_ (Model 1; Table S6; illustrated in Figure 1). Among partnered parents, the increase was from 9% to 54%. In the course of the study, 81 respondents lost their partner. Care was more frequently received when a parent lost their partner after T_0_ than a parent who remained unpartnered during the entire observation period.

The mean scores for the three variables on norms were approximately in the middle of the scale range (Table S7). Over time, the number of chronic diseases did not increase (B = 0.01; *p* > .05). Self-perceived poor health increased on average from 2.4 at T_0_ to 2.7 at T_6_, physical limitations from 1.5 to 2.1, and cognitive limitations from 1.1 to 1.5 (all *p* < .001). Averaged across the observations, 75% of unpartnered and 54% of partnered parents received care from one or more sources (partner – if applicable –, other informal, formal, private) other than their children.

### Predicting Intergenerational Care

Answering research question 1, we found that having more children increased the likelihood of receiving intergenerational care, both among unpartnered and partnered parents (Model 2; Table S8; estimated probabilities are illustrated in Figure S3). The models for a nonlinear and linear relationship were not far apart, and we continued the analyses with the linear relationship to decrease complexity. Among the unpartnered parents, the probability to receive care increased from 0.28 when having one child to 0.78 when having five or more children. For partnered parents, this was 0.14 and 0.42, respectively.

**Figure 2.**
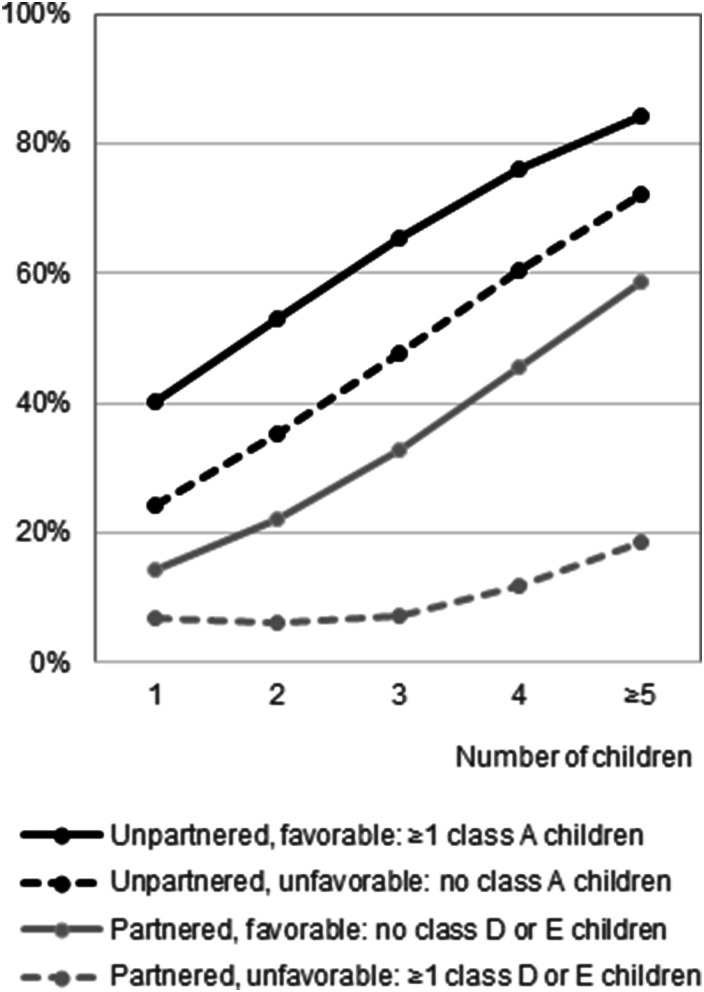
Predicted probabilities of receiving intergenerational care among older parents. *Notes:* Unpartnered: N_respondents_ = 196, N_observations_ = 1068; Partnered: N_respondents_ = 314, N_observations_ = 1791. Estimated marginal means based on Model 4 (Table S10). Controlled for time since T_0_ and having a daughter. To calculate the predicted probabilities, the choice of the least favorable option varies among partnered parents. When having one child, it is assumed that this child is in class E. For two children, one is in class D and one in E. For three children, two are in class D and one in E. For four and five children, two are in class D and one in E, and the other(s) are in any class.

To answer research question 2, we searched for the significance of having children in specific care potential latent classes. We do not discuss the intermediate results of all variants of Model 3 (Table S9) but focus on Model 4 (Table S10; predicted probabilities are illustrated in Figure 2). Among unpartnered parents (*N* = 196), having one or more children in high care potential class A (34% of unpartnered parents) increased the likelihood of receiving care by about 0.16 compared to when there was no child in class A. Having children in other classes, having one or more daughters and the interaction between the children’s gender and the parent’s gender did not contribute significantly to the prediction.

Among partnered parents (*N* = 314), having no children in classes D or E (54% of the partnered parents) increased the likelihood of receiving care. In this favorable circumstance, the probability increased from 0.14 when there was one child to 0.59 when there were five or more children – all in the high care potential class A or in the medium care potential classes B or C. In the unfavorable circumstance – when there were children in the low care potential classes D or E −, the probability increased from 0.07 when there was one child to 0.19 when there were five or more children. When a partnered parent had at least one daughter, the predicted probability was 0.25, compared to 0.19 when there were only sons; the interaction with the parent’s gender was not significant.

In Model 5 we included contextual factors (Table 1). Compared to Model 4, standard errors of the joint care potential parameters are generally larger, indicating a poorer fit. The model parameters are also less favorable. The effects of having a daughter increased. Mothers were more likely to receive intergenerational care than fathers. Parents with high income or educational level were less likely to receive intergenerational care; they were more likely to purchase care privately (bivariate results not shown). The unexpected negative effect of norms towards informal care cannot be explained and did not appear either in bivariate regression or robustness check (Table S11). The higher the filial expectations among unpartnered parents, the higher the likelihood that they received intergenerational care. Parents were more likely to receive intergenerational care as their age increased (indicated by age at baseline and time) and if they had physical and cognitive limitations. When partnered parents lost their partner, the estimated marginal mean of the probability for intergenerational care was 0.29, compared to 0.05 when the partner relationship continued. Finally, receiving care from another source increased the likelihood of receiving intergenerational care.

**Table 1.** Multilevel Logistic Regression on Whether Parents Received Intergenerational Care, Stratified by Partnership at T0 (Model 5).

	Unpartnered (N_respondents_ = 196, N_observations_ = 1068)				Partnered (N_respondents_ = 314, N_observations_ = 1791)			
	B	SE	*t*		B	SE	*t*	
Constant	−13.04	1.65	−7.9	***	−15.24	1.67	−9.1	***
Time-invariant predictors								
Number of children (1–5)	0.40	0.07	5.8	***	0.51	0.08	6.8	***
Relational care potential class								
A (high; one or more children vs. none)	0.94	0.18	5.2	***				
D (low; one child vs. none)					−0.68	0.19	−3.6	***
D (low; two or more children vs. none)					−0.82	0.24	−3.5	***
E (low; one or more children vs. none)					−1.55	0.30	−5.2	***
Whether having a daughter (vs. Only sons)	0.58	0.22	2.7	**	0.59	0.23	2.5	*
Age of parent (at baseline; 70–97)	0.11	0.02	6.2	***	0.12	0.02	6.6	***
Female (ref. Male)	0.01	0.28	0.0		0.47	0.16	2.9	**
Income (0.9–4.6 monthly in thousand Euro)	−0.37	0.11	−3.3	***	0.08	0.15	0.6	
Educational level (5–18 years)	−0.02	0.03	−0.5		−0.11	0.03	−3.8	***
Norms								
Norms towards informal care (1–5)	−0.22	0.10	−2.2	*	0.07	0.08	0.8	
Norms towards formal care (1–5)	−0.03	0.08	−0.3		−0.10	0.08	−1.2	
Filial responsibility expectation (1–5)	0.39	0.13	3.0	**	0.08	0.14	0.6	
Stepparent (ref. not)					−0.33	0.47	−0.7	
Time-variant predictors								
Time since baseline (0–10.7 years)	0.15	0.03	5.1	***	0.25	0.03	8.5	***
Care need								
Number of chronic diseases (0–4)	−0.08	0.08	−1.1		0.13	0.07	1.9	
Self-perceived poor health (1–4)	0.21	0.11	1.8		0.15	0.10	1.5	
Physical limitations (1–4)	0.66	0.13	5.2	***	0.45	0.11	4.1	***
Cognitive limitations (0–5)	0.20	0.05	3.7	***	0.09	0.05	1.8	
Lost the partner (ref. not)					2.14	0.22	9.5	***
Use of alternative care options								
Care from partner (ref. no)					0.15	0.19	0.8	
Care from other informal caregivers (ref. no)	0.37	0.18	2.1	*	0.92	0.19	4.7	***
Publicly paid care (ref. no)	0.19	0.20	1.0		0.46	0.18	2.5	*
Privately paid care (ref. no)	0.80	0.21	3.9	***	−0.21	0.19	−1.1	
Model parameters								
AIC corrected	5352.3				9698.8			
BIC	5386.9				9737.1			
*F*	13.9		***		19.5		***	
*df*_*1*_ *, df*_*2*_	18, 1049				23, 1767			

*p < .05; **p < .01; ***p < .001.

As a robustness check, we excluded the baseline observation to examine the predictive effects of joint care potential at later times. In general, the results (Table S11) are similar to those presented in Table 1. This suggests that the cross-sectional translation of joint care potential to care receipt is similar to the longitudinal translation.

## Discussion

At baseline, the majority of Dutch parents over the age of 70 did not receive care from their children, even among those without a partner. We observed an increase in the percentage of parents receiving care over ten years. This highlights the activation of intergenerational care potential as parents age, their health declines, and particularly after the loss of the partner. This finding underscores the role of adult children as a source of old-age security. Their care potential often remains dormant and is only activated when they perceive a clear, necessary, or urgent need for care from their aging parents.

### Intergenerational Care Potential and Care Receipt

We aimed to examine the extent to which joint intergenerational care potential translated into parent’s care receipt. Specifically, we looked at quantity (number of children) (research question 1) and quality (care potential classes) (research question 2). The answers to our research questions varied depending on whether the parents had a partner.

We first discuss parents without a partner. In answering research question 1, we found that the likelihood of care receipt increased linearly with the number of children: Each additional child increased the likelihood by 12 percentage points. Children may have complementary roles relative to siblings. For example, if a child is unwilling, unavailable, or unable to provide care at a particular time, another child may step in. Previously research (Grundy & Read, 2012; Sun, 2002) has also indicated that the number of children affects care receipt, but results varied on the exact number needed to have the effect. In answering research question 2, our results confirmed the literature-based expectation (Dykstra & Fokkema, 2011; Pillemer & Suitor, 2014) that having children with greater care potential – the *tight-knit* – increases the likelihood of receiving intergenerational care. Specifically, in addition to the number of children, having one or more children in the high care potential class further increased an unpartnered parent’s likelihood of receiving intergenerational care by about 16 percentage points. However, we also found that having one or more children in a medium care potential class did not further increase the likelihood of receiving care. For a parent with limited care need, it may not be necessary to have children with all the favorable characteristics. Help with administration, for example, does not always require geographical proximity. However, if care needs are more intensive or complex, it may be necessary and sufficient to have one child with all the favorable characteristics.

Among partnered parents, having an additional child increased the likelihood of receiving care by 11 percentage points (research question 1), but this effect seemed conditional: it only held if all children have medium or high care potential (research question 2). The results highlight the importance of the idea of joint care potential, meaning that a parent is more likely to receive intergenerational care if all children have some level of care potential (Tolkacheva et al., 2010). The negative effect of having one or more children in the low care potential classes – the *remote and staying in touch* and the *detached* children – almost entirely offset the positive effect of having a larger number of children. When all children have high or medium care potential, the probability of receiving intergenerational care increased significantly. Specifically, it was 7 percentage points higher for having one child and 40 percentage points higher for having five or more children. On average, this resulted in 24 percentage points increase in the likelihood of receiving care compared to the unfavorable circumstance (i.e., having one or more children with low care potential). Therefore, we add to the answer to the second research question that it did not matter whether children had medium or high care potential, as long as each child had at least medium care potential. Complementarity may thus arise as different children contribute at different times, increasing the likelihood of receiving care at each time. This effect is enhanced when there are more children with the favorable characteristics.

Combining the conclusions for both unpartnered and partnered parents, we find that the care potential of children plays a significant role. Caregiving often has a voluntary aspect, particularly when multiple children are present. Thus, the strength of the parent-child bond is reflected in caregiving behaviors. In cases of clear, urgent, or long-term care needs (e.g., in the event of the loss of a partner), having at least one child with high care potential becomes critical, because they are more likely to serve as a primary caregiver—a topic widely discussed in the parental care literature (Cicirelli, 1992; Hu et al., 2022). A child with high care potential is more likely to provide responsive and continuous care and be more resilient to caregiving challenges (Grigoryeva, 2017). Conversely, when care needs are less obvious or urgent, caregiving tends to occur more sporadically. For parents with all children having low care potential who have distant or even broken relationships with parents, the likelihood of receiving care is significantly low. This aligns with the depiction of family in the era of an aging society by Riley (1983, p. 439): “Relationships are no longer prescribed as strict obligations and must instead be earned—created and recreated by family members throughout their long lives.” Theoretically, we provide more insight into how the Intergenerational Solidarity Model (Bengtson & Roberts, 1991) works: both quantitative aspects (having more children) and qualitative aspects (specific combinations of the dimensions of opportunity, association, and function) are important for the likelihood of receiving care.

Traditionally, it is mainly daughters who provide care, not sons. We observed that having at least one daughter did not increase the probability of intergenerational care significantly, which is opposite to the findings of Batur et al. (2024). One possible reason for the small gender difference observed in this study might be gender is just a proxy for indicators of the care potential of a child such as contact frequency and emotional support. When we take these indicators into care potential, gender itself loses its impact. Another reason might be the fact that sons feel more obligated to provide care in the absence of daughters. We also found no interaction of children’s gender composition with their parent’s gender. From a parent’s perspective, the norms towards intergenerational care over other forms of care (e.g., formal care) might be irrespective of the gender of the children.

The translation of joint care potential also depends on the structural and social context. For example, the presence of a partner significantly impacts the translation from joint care potential to intergenerational care receipt. Unpartnered parents were more likely to receive intergenerational care than partnered parents, with a difference of about 25 percentage points. Moreover, among the partnered parents, those who lost their partner during the course of our study were much more likely to receive intergenerational care than those who did not. Swinkels et al. (2022) previously suggested that when a partner is present, a parent tends to receive personal care primarily from their partner and very rarely from their children. This might be due to factors such as affinity, intimacy, and geographic proximity with the partner. Children of unpartnered parents may provide care because they perceive living alone as a sign of vulnerability or feel a stronger sense of responsibility towards their lone parent. Thus, examining the extent of the translation of joint care potential into care receipt must take into consideration the presence of a partner.

### The Relevance of Care Need

We used comprehensive indicators to reveal care need. Being older, losing a partnership during the course of the study, and physical and cognitive impairments were associated with an increased likelihood of receiving intergenerational care. This highlights the complexity of “care need” in family studies. Simply using chronological age as an indicator of care need has been criticized (Grenier, 2020), as higher age does not necessarily imply an increased need for care. Additionally, using chronological age as a measure for care need reinforces the devaluation of older age. However, in our study, chronological age correlated with various care need indicators, and a higher chronological age did increase the likelihood of receiving intergenerational care. These findings suggest that higher chronological age may indicate a care need not captured by the other indicators we used. This could imply that children perceive older age as a signal to provide care to parents, regardless of the actual need. Children may also provide unsolicited care, irrespective of care need, as a way to show love, meet filial expectations, or reciprocate the support they received from their parents earlier in life (Leopold & Raab, 2013).

### Strengths and Limitations

A strength and novelty of this study is the integration of care potential at both the individual child and family levels in investigating parental care receipt. This approach has the advantage of demonstrating the interplay of various characteristics of individual relationships. Moreover, we were able to simultaneously describe multiple characteristics of multiple relationships within a family and to focus on the family configuration of intergenerational relationships. Finally, our study benefited from a powerful test of the (latent) nature of care potential with the prospective design, which included up to six follow-up observations spanning ten years.

We found that relational care potential indicators were on average fairly constant before and within our observation period. However, this average picture of stability masks that travel time, contact frequency, and support exchange do have the potential to change, which was visible by their own low correlations over time. Furthermore, additional indicators can be used to distinguish relational care potential. We suggest the availability of time (other care responsibilities and paid work), relational ambivalence (Fingerman et al., 2012) and care burden related relational difficulties (Offer & Fischer, 2018). Furthermore, the cohort change requires attention. van der Pas et al. (2007) showed that later born older adults had fewer children than the early born. Contact frequency and support exchange increased, but the decreased fertility rate might pose a threat to the joint care potential of the following cohorts.

We included data from phone and proxy interviews in our analyses to include frail parents, but this did come at the expense of data completeness. We lacked data on partners. A partnered parent without care need may receive intergenerational care as a by-product of their partner’s care need. For example, in response to a partner’s high care need, a child could do household tasks that benefit both parents. Therefore, we may have overestimated care receipt for partnered parents. However, given a partner’s primary role in care provision and the lower likelihood of receiving intergenerational care among partnered parents compared to unpartnered parents, we do not expect this to have had a large effect on our results.

## Conclusion

Intergenerational care potential could be further recognized and developed by families and social care practitioners to prolong the independent living of older parents. It is not only the number of children that counts. For unpartnered parents, the combination of favorable characteristics of intergenerational relationships mattered, rather than a single characteristic. Among partnered parents, it is required that all children have at least medium caregiving potential. Policy and practice should not assume that an older parent will receive care simply because there are multiple children or because there is a child living nearby.

## Supplemental Material

Supplemental Material - The Translation of Intergenerational Care Potential Into Care Receipt of Older Parents: A Prospective Study Supplemental Material for The Translation of Intergenerational Care Potential Into Care Receipt of Older Parents: A Prospective Study by Ying Shen, Theo G. van Tilburg, and Mariska van der Horst in Research on Aging
