# Supplementary material for: The Translation of Intergenerational Care Potential Into Care Receipt of Older Parents: A Prospective Study
Source: Res Aging. 2025 Mar 14;47(7-8):392–403. doi: 10.1177/01640275251326507 (PMC12102514; doi:10.1177/01640275251326507)
Supplement: Supplemental Material - The Translation of Intergenerational Care Potential Into Care Receipt of Older Parents: A Prospective Study [file sj-pdf-1-roa-10.1177_01640275251326507.pdf]

The translation of intergenerational care potential into care receipt of older parents:
A prospective study

Supplementary Material

Additional information on the analytic sample

Respondents born in 1940 or earlier, as they were invited to participate in three additional follow-up observations in addition to the regular observations (T_2-T_4; Supplementary Figure S1). We selected older adults who participated in at least the first follow-up observation (T_1) to examine the intergenerational care receipt prospectively. We excluded, both at T_0 and T_1, respondents (1) who were not living independently or were hospitalized, (2) who did not have any adult children, (3) whose partner was in an institution, (4) who were partnered but were not sharing a household with their partner, and (5) who had a new partner after baseline (T_0). These subgroups were excluded because the mechanisms of care receipt might be different from the respondents that were included in the trimmed dataset. The sample size of the omitted subgroups was not large enough to be treated as separate categories in this study.

There were six follow-up observations. Not all respondents participated in all follow-up observations because the individuals were deceased, ineligible due to illness, or refused to participate. Moreover, if respondents fell into one of the five exclusion categories after T_1, these observations were also omitted. This resulted in 2,859 observations among 510 respondents included in our final analyses (two to seven observations, M = 5.6; 44% had all seven observations).

The parents, 202 males and 308 females. had an average of 2.8 biological children (range 0–9). At baseline (T_0), twenty respondents had stepchildren from the current partnership, and three respondents had adoptive children. After T_0, 15 children died and some partnerships ended, affecting 23 stepchildren. We excluded 34 children who died before T_{-6}, 22 children who died between T_{-6} and T_0, and 34 former stepchildren from a partnership that ended before T_0.

Of the observations, 82 per cent of the interviews took place with respondents in person and 8 per cent by phone. In 10 per cent, a proxy was interviewed (often the partner or a child).

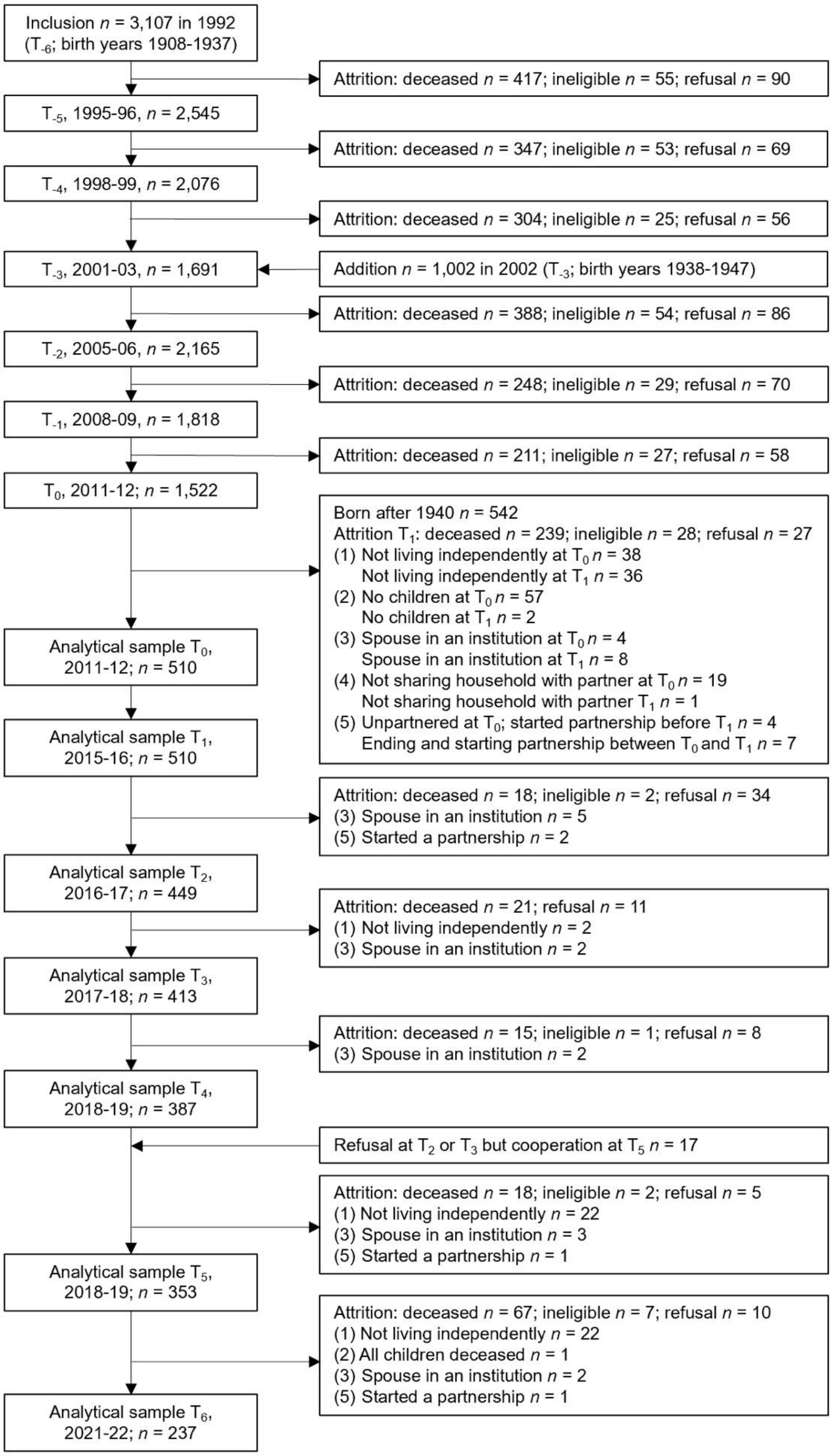

Figure S1. *Flowchart of respondent and observation selection*

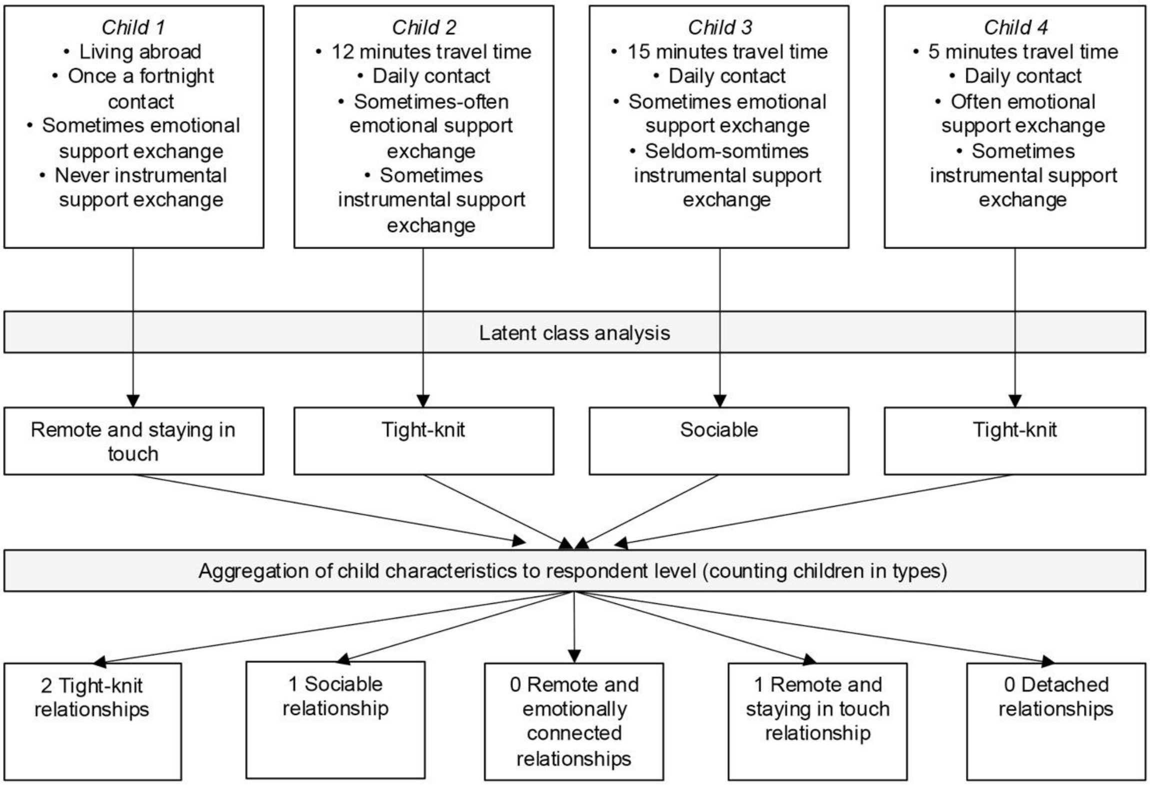

Figure S2. *An example of Latent Class Analysis and aggregation of child characteristics to respondent level*

Missing data at children's level

We primarily used information of children at baseline (T_0). However, children's information was collected in a section of the questionnaire where the parent's network was delineated by asking about individuals with whom there was significant and frequent contact at baseline [Anonymized for Review]. Because of abbreviated, aborted or substitute interviews, or because not all children were identified as network member, there are missing values for contact frequency (21%; Supplementary Table S1). Travel time and support exchange was only asked about the ten network persons with whom there was the most frequent contact, with about 33% missing values. Previous observations included the network delineation, and some included questions on relationships with all children. Data from preferably the last previous observation were used to impute missing values. Missing data at children's level after imputation were estimated with Full Information Maximum Likelihood imbedded in SPSS.

Table S1. *Children's data derivation from previous observations when missing at T_0 ($N_{relationships}$ = 1,496)*

Year	Travel time	Contact frequency	Received emotional support	Received instrumental support	Given emotional support	Given instrumental support
Missing	10(1)	0	47(3)	47(3)	47(3)	47(3)
1992-1993 (T_{-6})	33(2)	13(1)	34(2)	34(2)	34(2)	34(2)
1995-1996 (T_{-5})	24(2)	15(1)	35(2)	35(2)	35(2)	35(2)
1998-1999 (T_{-4})	25(2)	11(1)	31(2)	31(2)	31(2)	31(2)
2001-2003 (T_{-3})	102(7)	54(4)	57(4)	57(4)	57(4)	57(4)
2005-2006 (T_{-2})	91(6)	44(3)	78(5)	78(5)	78(5)	78(5)
2008-2009 (T_{-1})	230(15)	170(11)	215(14)	214(14)	215(14)	214(14)
2011-2012 (T_0)	981(66)	1189(79)	999(67)	1000(67)	999(67)	1000(67)

Notes: Numbers in brackets are percentages. We noticed that there was mismatch between contact frequency and support exchange, because care potential variables could be imputed from different years if they were missing at baseline. Thus, when contact with children was 'once a year or less' and data of support exchange were from earlier years, we do not accept data of support exchange but impute 1 ('never') instead.

Missing data at parent's level

Missing data of time-invariant variables at parent's level (at baseline) were mainly from filial responsibility expectations and norms towards care. As filial responsibility expectations were not asked at baseline, we derived answers from latest previous observations, or later observations if answers in earlier observations were missing. We did the same for norms towards care. Since filial responsibility expectations and norms towards care are deeply imbedded in daily life and children learn it through intergenerational interactions from a young age (Parrott & Bengtson, 1999), analyzing data from earlier or later observations should have relatively limited impact on the results.

Missing data of time-variant variables at observations at parent's level were mainly from cognitive impairments (10%). These missing values are mainly due to proxy interviews where the questions were not included. Cognitive functioning was imputed from the mean of the participant's previous and later observations (if available) to get a more accurate estimation. A small number of missing values were replaced by the sample mean.

Description of care potential characteristics

Table S2. *Descriptive statistics of care potential characteristics of children at T_0*

	N	%
Travel time		
0-9 minutes	326	22
10-19 minutes	335	22
21-29 minutes	117	8
30-39 minutes	168	11
40-49 minutes	107	7
About one hour	151	10
Between one and two hours	129	9
Two hours or more	153	10
Missing	10	1
Contact frequency		
Once per month or less often	181	12
Once a fortnight	203	14
Once a week	411	27
Few times a week	515	34
Everyday	186	12
Missing	0	0
Emotional exchange (mean)		
Never, or between never and seldom	104	7
Seldom	121	8
Between seldom and sometimes	194	13
Sometimes	414	28
Between sometimes and often	306	20
Often	322	22
Missing	35	2
Instrumental exchange (mean)		
Never	305	20
Never, or between never and seldom	191	13
Seldom	291	19
Between seldom and sometimes	302	20
Sometimes	233	16
Between sometimes and often, or often	139	9
Missing	35	2

Notes: $N_{relationships}$ = 1,496.

Dynamics in the care potential indicators

To explore the dynamics in the care potential indicators, we compared baseline indicators with the first and last available observation for the same intergenerational relationships, i.e., on average, these observations occurred seventeen years earlier and seven years later than baseline, respectively (Table S3). Mean travel time remained the same and mean contact frequency and characteristics of support exchange decreased between the first available observation and baseline. The effect sizes of time were moderate or weak. Support decreased further after baseline with the effect sizes of time remaining weak.

Table S3. *Pairwise t-test comparing baseline relational characteristics with those at the first and last available observation*

		Interval (years)		First available observation		Baseline				
	N	M	SD	M	SD	M	SD	*r*	*t*	Cohen's *d*
Travel time (0-1440 minutes)	971	16.9	5.0	47.6	128.8	51.4	120.9	0.35	-0.8	-0.03
Contact frequency (1-8)	1,179	16.9	4.9	6.6	1.2	6.3	1.2	0.44	9.6***	0.28
Emotional exchange (mean; 1-4)	981	16.6	5.1	3.2	0.7	3.1	0.7	0.39	2.1*	0.07
Instrumental exchange (mean; 1-4)	982	16.6	5.1	2.3	0.9	2.2	0.8	0.34	4.6***	0.15
		**Interval (years)**		**Baseline**		**Last available observation**				
	N	M	SD	M	SD	M	SD	*r*	*t*	Cohen's *d*
Travel time (0-1440 minutes)	792	7.2	2.5	52.5	122.6	52.3	127.5	0.66	0.1	0.00
Contact frequency (1-8)	1,014	7.2	2.5	6.2	1.2	6.3	1.3	0.56	-0.9	-0.03
Emotional exchange (mean; 1-4)	808	7.2	2.5	3.2	0.7	3.1	0.8	0.46	2.4*	-0.07
Instrumental exchange (mean; 1-4)	807	7.2	2.5	2.2	0.8	2.1	0.8	0.36	3.0**	0.11

Notes: $N_{relationships}$ = 1,496. Baseline observation is T_0. The first available observation varies between T_{-6} and T_{-1}. The last available observation can be T_1, T_2 or T_6.
* $p < 0.05$; ** $p < 0.01$; *** $p < 0.001$.

Model fit in Latent Class Analysis

Table S4. *Model fit statistics for relational care potential of children from one to eight classes*

Number of classes	Number of parameters	LL	BIC	AIC	L^2	df	*p*-value	Entropy R^2
1	21	-10373	20899	20787	2180	1418	<.001	1.00
2	26	-10123	20435	20297	1680	1413	<.001	0.56
3	31	-10070	20366	20202	1575	1408	0.001	0.57
4	36	-10031	20326	20135	1498	1403	0.04	0.57
5	41	-10003	20306	20088	1441	1398	0.21	0.56
6	46	-9992	20321	20077	1420	1393	0.30	0.56
7	51	-9973	20318	20047	1380	1388	0.55	0.56
8	56	-9964	20337	20039	1362	1383	0.65	0.56

Notes: $N_{relationships}$ = 1,496. LL = log likelihood; BIC = Bayesian Information Criterion, based on LL; AIC = Akaike Information Criterion, based on LL, L^2 = likelihood ratio chi-square goodness of fit test, df = degree of freedom. The LCA solution with five classes has the smallest BIC value. L^2 and corresponding *p*-values implied that an adequate overall goodness of fit starts with five classes. Entropy did not differ between models with multiple classes.

Description of relational care potential types

Table S5. *Five-class solution for relational care potential (column percentages)*

	A<br>Tight-knit<br>n = 240	B<br>Sociable<br>n = 485	C<br>Remote and emotionally connected<br>n = 351	D<br>Remote and staying in touch<br>n = 347	E<br>Detached<br>n = 73
Travel time					
0-9 minutes	49	39	2	1	10
10-19 minutes	41	38	5	4	27
21-29 minutes	5	11	7	6	10
30-39 minutes	5	9	17	13	11
40-49 minutes	0	2	15	12	7
About one hour	0	0	24	15	21
Between one and two hours	0	0	17	18	10
Two hours or more	0	0	13	31	5
Contact frequency					
Once per month or less	0	2	1	34	71
Once a fortnight	0	10	6	35	21
Once a week	2	36	39	26	8
Few times a week	55	43	44	5	0
Everyday	43	10	10	0	0
Emotional exchange					
Never, or between never and seldom	0	7	0	3	88
Seldom	0	15	0	13	12
Between seldom and sometimes	1	25	1	21	0
Sometimes	10	39	15	44	0
Between sometimes and often	37	13	31	15	0
Often	52	0	52	4	0
Instrumental exchange					
Never	1	13	15	40	86
Between never and seldom	0	14	12	22	8
Seldom	3	27	23	22	5
Between seldom and sometimes	28	24	25	10	0
Sometimes	30	18	17	4	0
Between sometimes and often, or often	38	5	7	1	0

Notes: N = 1,496. We set the two highest percentages of each indicator in boxes. We ordered classes by their count of advantages and disadvantages in four relational care potential indicators.

Longitudinal model of care receipt

Table S6. *Multilevel logistic regression of whether parents received intergenerational care on time (Model 1)*

	Unpartnered ($N_{respondents}$ = 196, $N_{observations}$ = 1,068)			Partnered ($N_{respondents}$ = 314, $N_{observations}$ = 1,791)		
	B	SE B	*t*	B	SE B	*t*
Intercept	-0.58	0.13	-4.5***	-2.27	0.13	-17.9***
Time (0-10 years)	0.14	0.02	5.8***	0.24	0.02	11.4***
Intercept				-0.09	0.20	-0.5
Time (0-10 years)				0.17	0.02	7.5***
Partner lost (no-yes)				2.24	0.16	14.1***

* $p < 0.05$; ** $p < 0.01$; *** $p < 0.001$.

Descriptive statistics of parents

Table S7. *Descriptive statistics, stratified by partnership at T_0*

	Unpartnered			Partnered		
	N	Mean	SD	N	Mean	SD
Characteristics of respondents at baseline						
Number of children (1-10)	196	3.1	1.6	314	2.9	1.4
Number of children in care potential class						
A (high; 0-7)	196	0.5	0.8	314	0.5	0.8
B (medium; 0-7)	196	0.9	1.0	314	1.0	1.2
C (medium; 0-5)	196	0.8	1.0	314	0.6	0.8
D (low; 0-6)	196	0.7	1.0	314	0.6	1.0
E (low; 0-4)	196	0.2	0.5	314	0.1	0.4
Whether having a daughter (ref. only sons)	196	0.79	0.41	314	0.81	0.39
Norms						
Norms towards informal care (1-5)	192	3.1	0.9	314	3.1	0.9
Norms towards formal care (1-5)	191	2.8	1.1	314	2.7	1.0
Filial responsibility expectations (1-5)	176	2.9	0.7	272	2.9	0.6
Control variables						
Female (ref. male)	196	0.87	0.33	314	0.44	0.50
Income (0.9-4.6 monthly in 1000 Euro)	195	2.6	0.9	312	2.5	0.5
Educational level (5-18 years)	196	9.3	3.2	314	10.3	3.3
Stepparent (ref. not)				314	0.06	0.24
Characteristics of respondents at T_0-T_6						
Care from children (ref. no)	1,068	0.52	0.50	1,791	0.27	0.44
Care need						
Number of chronic diseases (0-4) [a]	1,067	1.7	1.1	1,790	1.5	1.1
Self-perceived poor health (1-4) [a]	1,066	2.6	0.8	1,787	2.5	0.8
Physical limitations (1-4) [a]	1,068	2.0	0.9	1,790	1.7	0.8
Cognitive limitations (0-5) [a]	911	1.4	1.6	1,674	1.2	1.4
Age of parent (70-102)	1,068	83.7	5.8	1,791	81.4	4.9
Lost the partner (ref. not)				1,791	0.15	0.36
Use of alternative care options						
Care from partner (ref. no)				1,791	0.18	0.38
Care from other informal care givers (ref. no)	1,068	0.31	0.46	1,791	0.14	0.35
Publicly paid care (ref. no)	1,068	0.44	0.50	1,791	0.21	0.41
Privately paid care (ref. no)	1,068	0.29	0.46	1,791	0.23	0.42

Notes: $N_{respondents}$ = 510; $N_{observations}$ = 2,859. [a] Values are top-coded.

Table S8. *Multilevel logistic regression of whether parents received intergenerational care on number of children (non-linear and linear estimates), stratified by partnership at T_0 (Model 2)*

	Unpartnered ($N_{respondents}$ = 196, $N_{observations}$ = 1,068)			Partnered ($N_{respondents}$ = 314, $N_{observations}$ = 1,791)		
	B	SE	*t*	B	SE	*t*
Constant	-1.13	0.19	-5.9***	-1.20	0.17	-7.0***
Time	0.16	0.03	6.4***	0.27	0.02	12.1***
Number of children (ref. one)						
Two	2.29	0.26	8.9***	1.15	0.22	5.1***
Three	1.77	0.26	6.8***	0.59	0.22	2.7**
Four	1.40	0.23	6.0***	-0.29	0.21	-1.4
Five or more	0.77	0.22	3.4***	-0.33	0.20	-1.6
Model parameters	AIC corrected	4721.3		AIC corrected	8368.9	
	BIC	4756.0		BIC	8407.2	
	$F_{(5, 1062)}$	26.1***		$F_{(5, 1785)}$	41.5	***
	B	SE	*t*	B	SE	*t*
Constant	-1.49	0.17	-9.0***	-2.23	0.16	-14.2***
Time	0.16	0.03	6.4***	0.26	0.02	12.1***
Number of children (1-5)	0.55	0.05	10.2***	0.38	0.05	7.9***
Model parameters	AIC corrected	4718.2		AIC corrected	8343.1	
	BIC	4752.9		BIC	8381.5	
	$F_{(2, 1065)}$	64.4***		$F_{(2, 1788)}$	94.8***	

* $p < 0.05$; ** $p < 0.01$; *** $p < 0.001$.

Linearity of the association between care received from children and number of children

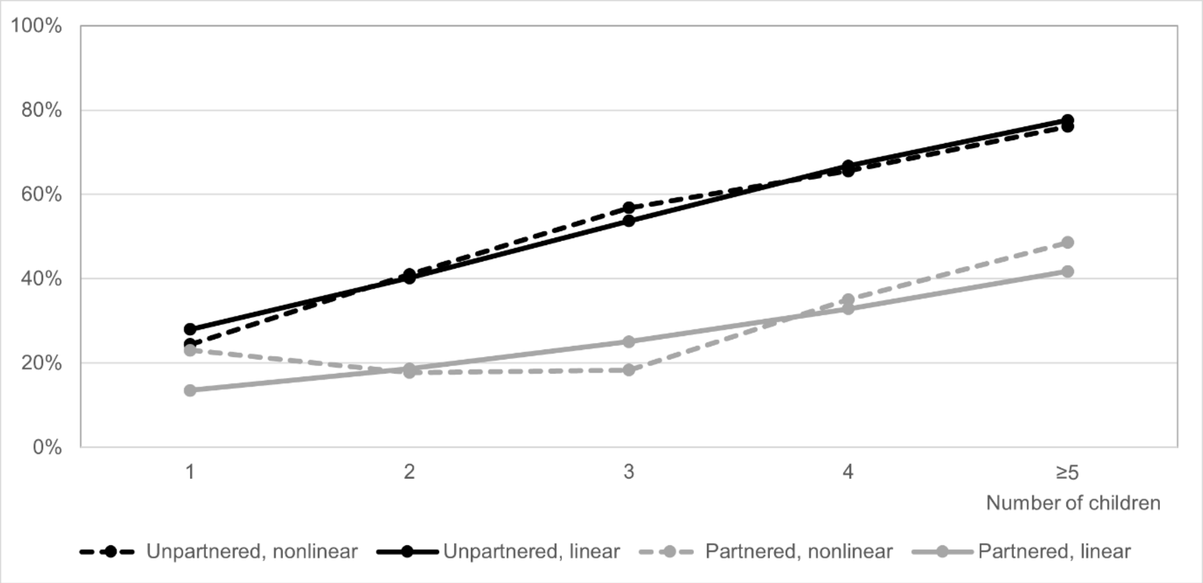

Figure S3. *Likelihood of care received from children by number of children*
Notes: Unpartnered: $N_{respondents}$ = 196, $N_{observations}$ = 1,068; Partnered: $N_{respondents}$ = 314, $N_{observations}$ = 1,791. Estimated marginal means based on Model 2 (Table S8). Controlled for time.

Predicting intergenerational care, without control variables

Table S9. *Fixed effects of having children in class(es), controlled for time and number of children (Models 3)*

Model	Having children in class(es)	Parameters	Unpartnered ($N_{respondents}$ = 196, $N_{observations}$ = 1,068)				Partnered ($N_{respondents}$ = 314, $N_{observations}$ = 1,791)			
			F	df_1	df_2	*p*	*F*	df_1	df_2	*p*
3a	A	A	24.1	1	1064	0.000	2.3	1	1787	0.126
3b	B	B	1.5	1	1064	0.214	3.8	2	1786	0.023
3c	C	C	0.1	2	1063	0.939	0.4	2	1786	0.667
3d	D	D	2.9	2	1063	0.057	18.7	2	1786	0.000
3e	E	E	0.0	1	1064	0.980	12.6	1	1787	0.000
3f	A + B	A	25.9	1	1062	0.000				
		B	2.5	2	1062	0.083				
3g	A + C	A	24.9	1	1062	0.000				
		C	0.6	2	1062	0.559				
3h	A + D	A	20.5	1	1062	0.000				
		D	1.1	2	1062	0.326				
3i	A + E	A	24.6	1	1063	0.000				
		E	0.6	1	1063	0.441				
3j	D + E	D					19.2	2	1785	0.000
		E					13.6	1	1785	0.000
3k	D + E + A	D					19.8	2	1784	0.000
		E					15.0	1	1784	0.000
		A					1.7	1	1784	0.189
3l	D + E + B	D					17.4	2	1783	0.000
		E					13.5	1	1783	0.000
		B					1.1	2	1783	0.350
3m	D + E + C	D					20.5	2	1783	0.000
		E					14.8	1	1783	0.000
		C					1.7	2	1783	0.176

Notes: The optimal Model 3 for unpartnered parents is Model 3a, and for partnered parents, it is Model 3j.

Table S10. *Multilevel Logistic regression on whether parents received intergenerational care, stratified by partnership at T_0 (Model 4)*

	Unpartnered ($N_{respondents}$ = 196, $N_{observations}$ = 1,068)			Partnered ($N_{respondents}$ = 314, $N_{observations}$ = 1,791)		
	B	SE	*t*	B	SE	*t*
Constant	-2.30	0.23	-9.8***	-3.93	0.24	-16.6***
Time-invariant predictors						
Number of children (1-5)	0.52	0.06	9.1***	0.54	0.06	9.0***
Relational care potential class						
A (high; one or more children vs. none)	0.73	0.15	5.0***			
D (low; one child vs. none)				-0.65	0.15	-4.3***
D (low; two or more children vs. none)				-1.03	0.19	-5.5***
E (low; one or more children vs. none)				-0.80	0.22	-3.7***
Whether having a daughter (vs. only sons)	-0.16	0.17	-1.0	0.38	0.18	2.1*
Time-variant predictors						
Time since baseline (0-10.7 years)	0.17	0.03	6.4***	0.27	0.02	12.6***
Model parameters						
AIC corrected		4755.6			8483.4	
BIC		4790.3			8521.7	
F		36.1***			39.4***	
df1, df2		4, 1063			6, 1784	

* $p < 0.05$; ** $p < 0.01$; *** $p < 0.001$.

Robustness check

Table S11. *Multilevel logistic regression of whether parents received intergenerational care, without baseline observation, stratified by partnership at T_0*

	Unpartnered ($N_{respondents}$ = 196, $N_{observations}$ = 872)					
	Model 4			Model 5		
	B	SE	*t*	B	SE	*t*
Constant	-2.43	0.35	-7.0***	-13.82	1.89	-7.3***
Time-invariant predictors						
Number of children (1-5)	0.51	0.06	8.2***	0.39	0.08	5.1***
Relational care potential class						
A (high; one or more children vs. none)	0.72	0.16	4.4***	0.95	0.20	4.7***
D (low; one child vs. none)						
D (low; two or more children vs. none)						
E (low; one or more children vs. none)						
Whether having a daughter (vs. only sons)	-0.32	0.19	-1.7	0.42	0.24	1.7
Age of parent (at baseline; 70-97)				0.11	0.02	5.7***
Female (ref. male)				0.06	0.31	0.2
Income (0.9-4.6 monthly in thousand Euro)				-0.28	0.12	-2.3*
Educational level (5-18 years)				-0.04	0.04	-1.1
Norms						
Norms towards informal care (1-5)				-0.19	0.11	-1.7
Norms towards formal care (1-5)				-0.01	0.09	-0.1
Filial responsibility expectations (1-5)				0.33	0.14	2.3*
Stepparent (ref. not)						
Time-variant predictors						
Time since baseline (0-10.7 years)	0.21	0.05	4.4***	0.24	0.05	4.5***
Care need						
Number of chronic diseases (0-4)				-0.12	0.08	-1.4
Self-perceived poor health (1-4)				0.26	0.12	2.1*
Physical limitations (1-4)				0.61	0.14	4.4***
Cognitive limitations (0-5)				0.23	0.06	3.9***
Lost the partner (ref. not)						
Use of alternative care options						
Care from partner (ref. no)						
Care from other informal caregivers (ref. no)				0.36	0.19	1.8
Publicly paid care (ref. no)				0.25	0.22	1.1
Privately paid care (ref. no)				0.82	0.23	3.6***
Model parameters						
AIC corrected		3876.5			4366.7	
BIC		3905.0			4395.1	
F		26.3***			11.1***	
df1, df2		4, 867			18, 853	

* $p < 0.05$; ** $p < 0.01$; *** $p < 0.001$.

Table S11. *(continued)*

	Partnered ($N_{respondents}$ = 314, $N_{observations}$ = 1,477)					
	Model 4			Model 5		
	B	SE	*t*	B	SE	*t*
Constant	-3.04	0.28	-11.0***	-14.41	1.79	-8.1***
Time-invariant predictors						
Number of children (1-5)	0.49	0.06	8.0***	0.46	0.08	5.6***
Relational care potential class						
A (high; one or more children vs. none)						
D (low; one child vs. none)	-0.49	0.15	-3.3***	-0.41	0.19	-2.1*
D (low; two or more children vs. none)	-0.99	0.19	-5.2***	-0.88	0.25	-3.5***
E (low; one or more children vs. none)	-0.81	0.22	-3.7***	-1.81	0.32	-5.6***
Whether having a daughter (vs. only sons)	0.33	0.18	1.8	0.57	0.23	2.5*
Age of parent (at baseline; 70-97)				0.12	0.02	5.7***
Female (ref. male)				0.42	0.17	2.5*
Income (0.9-4.6 monthly in thousand Euro)				0.34	0.16	2.2*
Educational level (5-18 years)				-0.11	0.03	-3.8***
Norms						
Norms towards informal care (1-5)				0.04	0.09	0.4
Norms towards formal care (1-5)				-0.05	0.08	-0.6
Filial responsibility expectations (1-5)				0.12	0.15	0.8
Stepparent (ref. not)				-0.65	0.55	-1.2
Time-variant predictors						
Time since baseline (0-10.7 years)	0.16	0.03	4.9***	0.13	0.04	2.9**
Care need						
Number of chronic diseases (0-4)				0.18	0.07	2.5*
Self-perceived poor health (1-4)				0.10	0.10	1.0
Physical limitations (1-4)				0.45	0.12	3.8***
Cognitive limitations (0-5)				0.11	0.05	2.0*
Lost the partner (ref. not)				2.09	0.22	9.7***
Use of alternative care options						
Care from partner (ref. no)				0.16	0.20	0.8
Care from other informal caregivers (ref. no)				1.07	0.20	5.4***
Publicly paid care (ref. no)				0.47	0.19	2.4*
Privately paid care (ref. no)				-0.10	0.19	-0.5
Model parameters						
AIC corrected		6676.8			7745.5	
BIC		6708.5			7777.1	
F		18.2***			15.6***	
df1, df2		6, 1470			23, 1453	

* $p < 0.05$; ** $p < 0.01$; *** $p < 0.001$.

References

Parrott, T. M., & Bengtson, V. L. (1999). The effects of earlier intergenerational affection, normative expectations, and family conflict on contemporary exchanges of help and support. *Research on Aging*, *21*(1), 73-105. https://doi.org/10.1177/0164027599211004

[Anonymized for Review]
